# Retinoid acid-induced *microRNA-31-5p* suppresses myogenic proliferation and differentiation by targeting *CamkIIδ*

**DOI:** 10.1186/s13395-017-0126-x

**Published:** 2017-05-11

**Authors:** Bo Liu, Chao Liu, Wei Cong, Nan Li, Nan Zhou, Yi Tang, Chao Wei, Han Bai, Ying Zhang, Jing Xiao

**Affiliations:** Department of Basic Oral Sciences, College of Stomatology, Dalian Medical University, Dalian, 116044 People’s Republic of China

**Keywords:** Retinoic acid, Tongue, miRNA, Myogenesis, CaMKIIδ

## Abstract

**Background:**

We previously reported that Wnt5a/CaMKIIδ (calcium/calmodulin-dependent protein kinase II delta) pathway was involved in the embryonic tongue deformity induced by excess retinoic acid (RA). Our latest study found that the expression of *miR-31-5p*, which was predicted to target the 3′UTR of *CamkIIδ*, was raised in the RA-treated embryonic tongue. Thus, we hypothesized that the excess RA regulated Wnt5a/CaMKIIδ pathway through *miR-31-5p* in embryonic tongue.

**Methods:**

C2C12 myoblast line was employed as an in vitro model to examine the suppression of *miR-31-5p* on *CamkIIδ* expression, through which RA impaired the myoblast proliferation and differentiation in embryonic tongue.

**Results:**

RA stimulated the expression of *miR-31-5p* in both embryonic tongue and C2C12 myoblasts. Luciferase reporter assay confirmed that the 3′UTR of *CamkIIδ* was a target of *miR-31-5p. MiR-31-5p* mimics disrupted *CamkIIδ* expression, C2C12 proliferation and differentiation as excess RA did, while *miR-31-5p* inhibitor partially rescued these defects in the presence of RA.

**Conclusions:**

Excess RA can stimulate *miR-31-5p* expression to suppress *CamkIIδ*, which represses the proliferation and differentiation of tongue myoblasts.

**Electronic supplementary material:**

The online version of this article (doi:10.1186/s13395-017-0126-x) contains supplementary material, which is available to authorized users.

## Background

Retinoic acid (RA) commonly refers to 11-cis-retinaldehyde and all-trans retinoic acid, the active metabolites of the natural nutrient vitamin A (retinol). These metabolites of vitamin A can form complexes with nuclear RA receptors (RARs) or retinoid X receptors (RXRs) and then, bind to the RA response element (RARE) in the enhancers of target genes to regulate their transcriptions [1, 2]. Although RA signaling plays an essential role in the morphogenesis and cell differentiation, excessive RA triggers teratogenic responses and birth defects [3–7]. Our previous study reported that RA administration could cause tongue deformity by disrupting the proliferation and differentiation of embryonic tongue muscles, especially the genioglossus. Wnt5a/calcium/calmodulin-dependent protein kinase II delta (CaMKIIδ) pathway was involved in the RA-induced myogenic abnormalities of tongue [8].

CaMKII is a ubiquitously expressed serine/threonine kinase and presents in four isoforms whose distribution are displayed in a cell-type specificity. CaMKIIα and β are mainly expressed in neural tissues, while CaMKIIγ and δ are the predominant isoforms in non-neural tissues [9]. As the predominant isoform in all three types of muscles, CaMKIIδ affects muscle differentiation and function in multiple levels. In embryonic stages, CaMKIIδ phosphorylates HDAC4 and HDAC5 to enhance—their nuclear exports and release—MEF2, which promotes the myogenic differentiation [10, 11]. During the post-natal period, pathological activation or transgene of *CamkIIδ* contributes to arrhythmias and heart failure [12–14]. Consistently, mouse lacking CaMKIIδ shows the resistance to pathological cardiac hypertrophy [15]. Because of the location in the cytoplasmic side of sarcoplasmic reticulum and nucleus of cardiomyocytes [16], CaMKIIδ is suggested to play a critical role in Ca^2+^ homeostasis for action potential and contraction. Moreover, recent studies discovered that the phosphorylation of histone by CaMKIIδ triggered mRNA alternative splicing in cardiomyocytes [15, 17]. However, except the reports that altered intracellular Ca^2+^ concentration and increased reactive oxygen species (ROS) are the activators for CaMKIIδ [18], few studies concern on the mechanisms controlling CaMKIIδ expression.

MicroRNAs (miRs) represent the RNAs constituted by 22–26 nucleotides, which regulate gene expression on post-transcription level. The miRs exclusively or preferentially expressed in muscles, including *miR-1*, *miR-133*, *miR206*, *miR-208*, *miR486*, and *miR499*, are defined as “muscle-specific” or “muscle enriched” miRs which play crucial roles in myogenesis and regeneration [19, 20]. Additionally, miRs more than the muscle-specific and enriched groups, such as *miR-185*, *miR-203*, *miR-214*, and *miR-378*, are also involved in myogenesis and muscle function [21–24]. The genes targeted by miRs during myogenesis and muscle regeneration cover multiple families of growth factors, receptors, signaling transducers, and transcription factors, indicating comprehensive regulations and complicated net effects of miRs on muscle development and function [25, 26]. However, if miRs in tongue muscles play the same roles as they do in the muscles of the limb and heart and if RA-induced tongue deformity and/or the CaMKIIδ down-regulation are mediated by miRs require to be elucidated.

## Methods

### Mice

According to the guidelines of the Ethics Committee of the Dalian Medical University, all procedures using mice in the present study strictly followed the protocol (L2014034) and ethical guidelines approved by the Animal Management Committee of Dalian Medical University. The day in which vaginal plug was detected in ICR female mice was designated as embryonic day 0.5 (E0.5). All pregnant mice were randomly divided into treatment and control groups at E10.5. The treatment group was intragastrically administrated with all-trans retinoic acid dissolved in edible oil (RA, Sigma-Aldrich, St. Louis, MO) at a dose of 100 mg/kg, while the control group was only given edible oil. The embryonic tongues were harvested at E14.5.

### Cell lines

Both C2C12 and 293 T cell lines were purchased from the Shanghai Institute of Cell Biology, Chinese Academy of Sciences (Shanghai, China). Growth medium (GM) for cell culture contained high-glucose Dulbecco’s Modified Eagle’s Medium (DMEM, Gibco, Carlsbad, CA, USA) with 10% fetal bovine serum (Gibco, Carlsbad, CA, USA). To induce myogenic differentiation, GM was replaced by differentiation medium (DM) containing high-glucose DMEM supplemented with 2% horse serum (HyClone, Logan, UT, USA) after C2C12 cells reached 80~90% confluence. RA supplement in medium was prepared as previously described [27]. All media were changed every 2 days.

### RNA extraction and quantitative real-time PCR (Q-PCR)

Total RNA was extracted from embryonic tongue or cultured cells with TRIzol Reagent (Takara, Japan). High-Capacity cDNA Reverse Transcription Kit (4366596, Applied Biosystems, CA, USA) was used for the reverse transcriptions of *miR-31-5p* by using specific miRNA primers (000185, Applied Biosystems, CA, USA) with U6 as an internal control (001973, Applied Biosystems, CA, USA). Then, the TaqMan®Universal Master Mix II Kit (no UNG; 4440040, ABI) was used for Q-PCR on ABI 7500 Fast Real-Time PCR System. Messenger RNA was reversely transcribed into complementary DNA (cDNA) with the PrimerScript RT regent Kit gDNA Eraser (DRR047A, Takara, Japan), and Q-PCR was performed by using SYBR® Premix Ex Taq^TM^ II (Perfect Real Time) Kit (DRR820A, Takara, Japan) on a Dice Real Time System Thermal Cycler (TP800, Takara, Japan) with GAPDH as an internal control. The sequences of primers were shown in Additional file 1: Table S1.

### Cell transfection

All the RNA oligonucleotides in this study (miRNA mimics, miRNA inhibitor, and siRNAs) were obtained from GenePharma (Suzhou, China). RNA oligonucleotides’ sequences are listed in Additional file 2: Table S2. The full length of *CamkIIδ* coding sequence was cloned into pcDNA3.1 and driven to be expressed by CMV promoter (GenePharma, Suzhou, China). For proliferation, cells were seeded into 96 or 24-well plates before transfection and transfected with Lipofectamine 3000 reagent (Invitrogen, Carlsbad, CA, USA) according to the manufacturer’s instruction when reached approximately 30% confluence. For myogenic differentiation assay, C2C12 cells were seeded in 12-well plates and transfected when reached 70% confluence. RNA oligonucleotides applied in cell transfection were described in Additional file 2: Table S2.

### Luciferase reporter assay

The 3′UTR of mouse *CamkIIδ* was cloned into pmirGLO vector (Promega, Madison, WI, USA) with Xhol and Sall at the 3′ end of the luciferase gene to construct the luciferase reporter plasmid *pmirGLO-CamkIIδ-WT*. The mutagenesis of 3′UTR of *CamkIIδ* was achieved by the QuickChange® Site-Directed Mutagenesis Kit (Stratagene). The seed sequences of *pmirGLO-CamkIIδ-WT* and *pmirGLO-CamkIIδ-MUT* were listed in the Additional file 3: Table S3. For the reporter assay, HEK293T cells were seeded in a 96-well plate and then co-transfected with 50 nM of *miR-31-5p* mimic, Duplex negative control (Duplex NC) or 100 ng of vectors per well. After 24 h of transfection, the activities of Firefly and Renilla luciferases were measured with a Dual-Glo Luciferase Assay System (Promega, Madison, WI, USA) according to the manufacturer’s instructions.

### Proliferation assay

For Cell Counting Kit-8 (CCK-8) assay, C2C12 cells were seeded at 5 × 10^3^ cells per well in 96-well plates and incubated with or without RA for 0, 24, 36, and 48 h after transfection. Cell proliferation was assessed by using Cell Counting Kit-8 (CCK-8, Dojin Laboratories, Kumamoto, Japan) following the manufacturer’s protocol. For EdU assay, C2C12 cells were seeded in 24-well plates and incubated under the standard conditions for 24 h. After incubation with 50 μM of 5-ethynyl-2′-deoxyuridine (EdU) from Proliferation Assay Kit (Ribobio, Guangzhou, China) for 2 h, cells were fixed and stained for EdU as described in the manufacturer’s protocol. The cell nuclei were counter-stained with Hoechst 3342 for 30 min and detected by fluorescence microscopy (DP72, Olympus, Japan).

### Immunocytochemistry

C2C12 cells in 24-well plates were fixed with 4% formaldehyde for 30 min and washed three times in PBS for 5 min each time. The fixed cells were incubated with 0.5% Triton X-100 in PBS for 10 min, 3% H_2_O_2_ for 10 min, the primary antibodies against myosin (MAB-0124, Maixin, Fuzhou, China) and CaMKIIδ (ab105502, Abcam, Cambrige, UK) overnight at 4 °C, and then, the secondary antibodies (Alexa Fluor 594, a11037, Invitrogen, Carlsbad, CA, USA) for 45 min before counterstained with 4′, 6-diamidino-2-phenylindole dihydrochloride (DAPI, D9542, Sigma, St. Louis, MO, USA) for 10 min. Images from at least three regions in each well were captured by a fluorescence microscope (DP72, Olympus, Japan). The fusion index was calculated as the ratio of the number of nuclei in myosin-positive myotubes with two or more nuclei to the total nuclei number.

### Western blotting

Tissues or cells were lysed in ice-cold RIPA buffer (50 mM Tris-HCl, 150 mM NaCl, 0.1% SDS, 1% NP-40, 0.25% sodium dexycholate, and 1 mM EDTA, pH8.0) with freshprotease/phosphatase inhibitor cocktail (Boster, Wuhan, China) for 30 min on ice and centrifuged at 16,000 g at 4 °C for 15 min. The protein concentration in supernatant was measured by a BCA protein assay kit (Boster, Wuhan, China). Proteins in the supernatant were separated by SDS-PAGE (Bio-Rad, Richmond, CA, USA) and then, transferred to PVDF membranes (Bio-Rad, Richmond, CA, USA) followed by the blockage in 5% skim milk for 2 h at room temperature. After the membranes were incubated with primary antibodies at 4 °C overnight, HRP-conjugated secondary antibodies were incubated with membrane for 1 h at room temperature. The enhanced chemiluminescence substrate (Boster, Wuhan, China) was applied for color development. The primary antibody against CaMKIIδ was purchased from Abcam (ab105502, Abcam, Cambrige, UK), the antibody against myosin from Millipore (05–716, monoclonal mouse IgG specific for different myosin heavy chain, Millipore Corporation, Billerica, MA, USA), and the antibody against glyceraldehyde 3-phosphate dehydrogenase (GAPDH) from Santa Cruze Biotechnology (sc-25778, Santa Cruz Biotechnology, Santa Cruz, CA, USA).

### Statistics

The results from at least three independent repeated experiments were collected for Student’s *t* test, and the statistical consequences were expressed as the means ± SD. Only when the *p* value was less than 0.05, the statistical difference was regarded to be significant.

## Results

### *MiR-31-5p* could directly target the 3′UTR of *CamkIIδ*

**Fig. 1:**
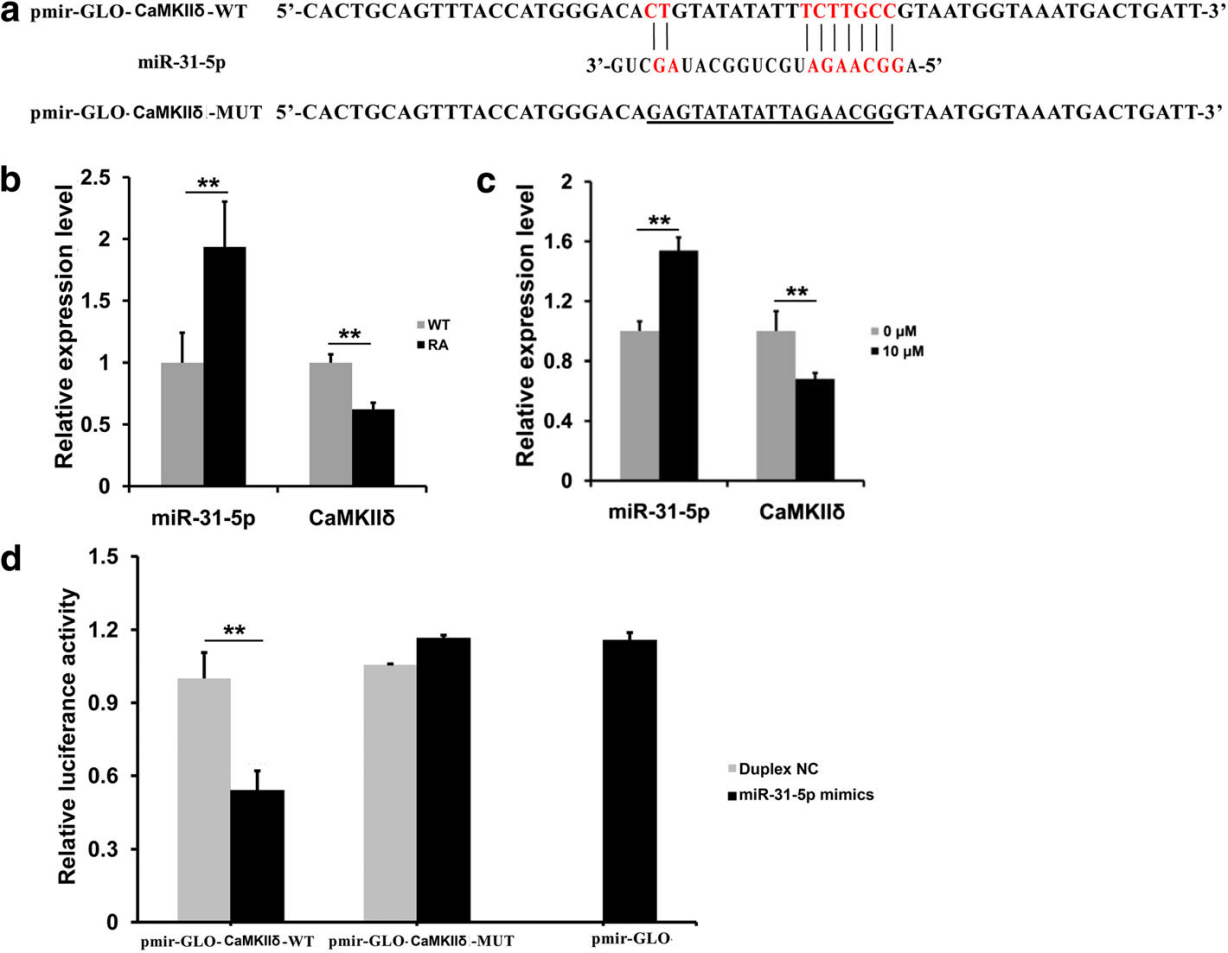
*MiR-31-5p* was able to interact with *CamkIIδ* directly. **a** The schematic diagram of luciferase reporter construct (*pmir-GLO-CaMKIIδ-WT*) and mutated reporter construct (*pmir-GLO-CaMKIIδ-MUT*). The sequences predicted by TargetScan for imperfect binding between mouse *miR-31-5p* (*mmu-miR-31-5p*) and 3′ UTR of *CamkIIδ*, and the vector sequences of WT (*pmir-GLO-CaMKIIδ-WT*) and mutant (*pmir-GLO-CaMKIIδ-MUT*) designed for luciferase activity assay. **b**, **c** Excess RA up-regulated *miR-31-5p* level and inhibited *CamkIIδ* expression in both mouse embryonic tongue and C2C12 cells. In vivo, the expression of *miR-31-5p* was up-regulated at E14.5 in the tongue of RA-treated mouse, nevertheless, the expression of *CamkIIδ*down-regulated at E14.5. In vitro, the relative expression of *miR-31-5p* was up-regulated and *CamkIIδ*was inhibited in the C2C12 myoblasts after 24 h supplemented with 10 μM RA. **d** Direct interaction between *miR-31-5p* and *CamkIIδ.* Plasmids of *pmir-GLO-CaMKIIδ-WT*, *pmir-GLO-CaMKIIδ-MUT*, and *pmir-GLO* were co-transfected into 293 T cells with duplex NC and *miR-31-5p* mimics. Luciferase activities were detected about 24 h after transfection. *MiR-31-5p* but not duplex NC, significantly inhibited the luciferase activity of the *pmir-GLO-CaMKIIδ-WT*; in contrast, it did not inhibit the activities of *pmir-GLO-CaMKIIδ-MUT* and *pmir-GLO*. (The difference time points in **b** and **c** were normalized by *GAPDH* expression as inner control; *P < 0.05, **P < 0.01; *pmir-GLO* in **d** was the empty vector working as inner control)

Our latest study found that several non-muscle specific or rich miRs, including *miR-31-5p*, gave rise to greatly drastically changed expression in the E14.5 RA-treated tongue [28]. All of the bioinformatics analysis with TargetScan, MiRanda, and MicroCosm predicted a complementary matching between a fragment of sequence in *miR-31-5p* and the 3′UTR of *CamkIIδ* (Fig. 1a). Q-PCR revealed that in the E14.5 RA-treated tongue, the rise of *miR-31-5p* expression was coincided with the fall of *CamkIIδ* transcription (Fig. 1b). Similar to the in vivo coincidence, RA supplement into the GM of C2C12 myoblasts also elevated the in vitro expression of *miR-31-5p*, but suppressed *CamkIIδ* expression (Fig. 1c). To validate the direct target binding between *miR-31-5p* and *CamkIIδ*, luciferase reporter assay was conducted in HEK 293 T cells. When co-transfected with *pmir-GLO-CaMKIIδ-WT*, *miR-31-5p* mimics was able to reduce luciferase activity compared with the duplex NC (Fig. 1d). In contrast, *miR-31-5p* mimics had no impact on the luciferase activity of *pmir-GLO-CaMKIIδ-MUT* as well as the control vector, *pmir-GLO* (Fig. 1d). These results indicated that the 3′UTR *CamkIIδ* could work as a direct binding target of *miR-31-5p*.

### *MiR-31-5p* mimics impaired *CamkIIδ* expression and C2C12 proliferation

**Fig. 2:**
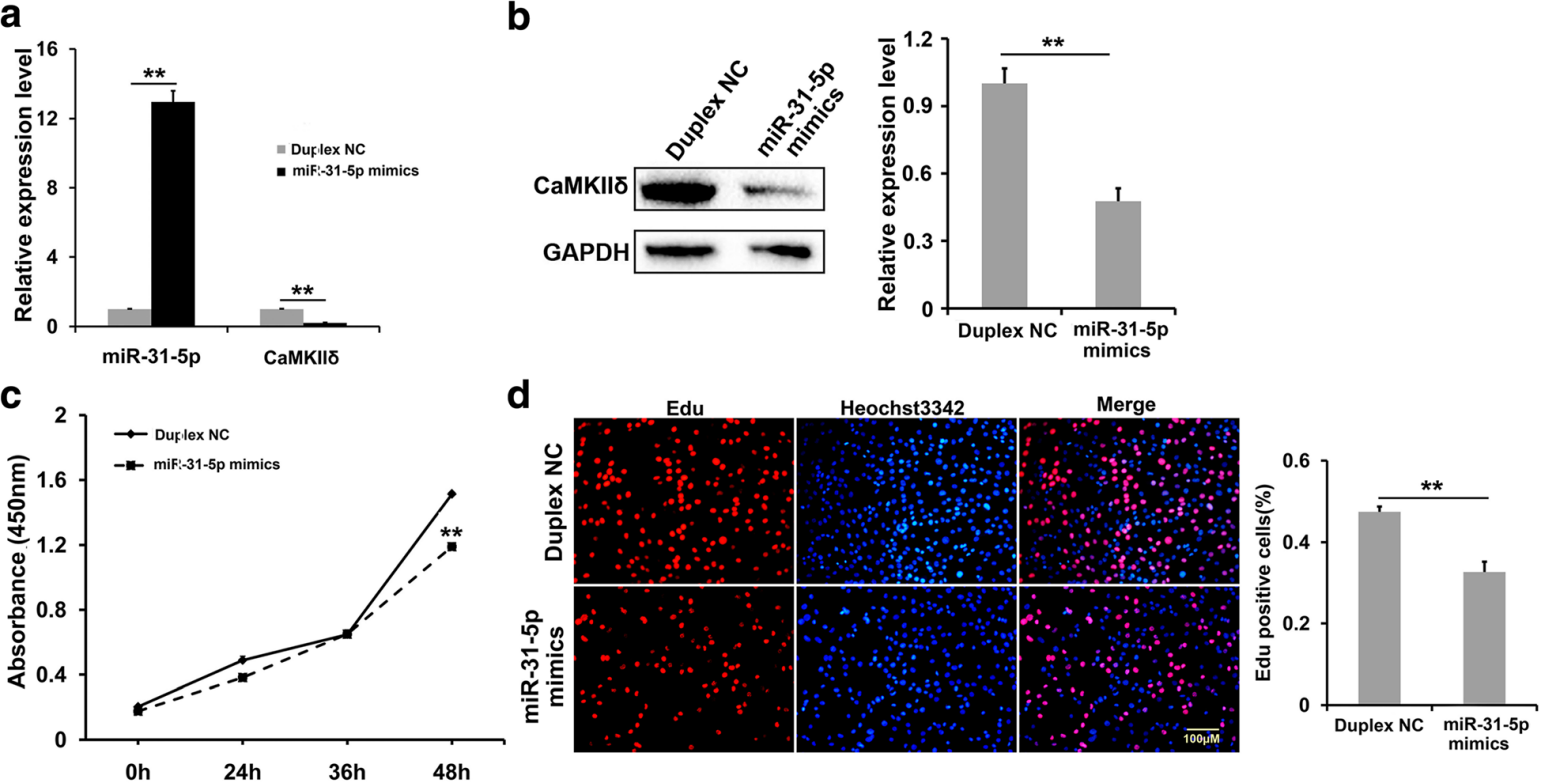
The effects of *miR-31-5p* over-expression on the proliferation of C2C12 myoblasts. **a** qRT-PCR analysis of the mRNA expression of *miR-31-5p* and *CamkIIδ. MiR-31-5p* mimics and duplex NC were transfected into C2C12 myoblasts, respectively. The relative expression level of *miR-31-5p* was 13-folds compared with duplex NC, while the *CamkIIδ* expression level decreased to 20.7% in cells transfected with *miR-31-5p* mimics as in duplex NC cells. **b** Western Blotting and statistical analysis for CaMKIIδ expression. Protein of CaMKIIδ was decreased 53.6% after 48 h of transfection of *miR-31-5p* compared with duplex NC trasfection. **c** CCK-8 assay for*miR-31-5p* affect the proliferation of C2C12 myoblasts. After 48 h transfection of *miR-31-5p* mimics, the number of C2C12 cells increased much slower than in control group, especially at 48 h. **d** Edu assay for the proliferation ratio of C2C12 myoblasts. The statistical assay confirmed the transfection of *miR-31-5p* mimics resulted in a decrease of Edu labeling cells to 60% of duplex NC group (**P < 0.01)

In order to address the causal relationship between *miR-31-5p* elevation and *CamkIIδ* down-regulation, the *miR-31-5p* mimics and duplex NC were transfected into C2C12 cells cultured in GM, respectively. In Q-PCR, the mimic-transfecting group exhibited a dramatic increase of *miR-31-5p* expression and a remarkable decrease of *CamkIIδ* expression, verifying the validity of *miR-31-5p* mimics, as well as the suppression on *CamkIIδ* (Fig. 2a). Western blotting and the statistical assay showed that the protein level of CaMKIIδ in *miR-31-5p* mimic-transfecting group was comparable to the half of that in duplex NC group (Fig. 2b). These results indicated that *miR-31-5p* suppressed *CamkIIδ* expression in myoblasts. Furthermore, C2C12 proliferation was also slowed down by the *miR-31-5p* mimic transfection in 48 h (Fig. 2c) because of the reduced proliferating percentage shown in EdU assay (Fig. 2d). Hence, the elevated *miR-31-5p* expression not only repressed *CamkIIδ* expression but also inhibited C2C12 proliferation.

### *MiR-31-5p* inhibitor rescued C2C12 proliferation disrupted by RA

**Fig. 3:**
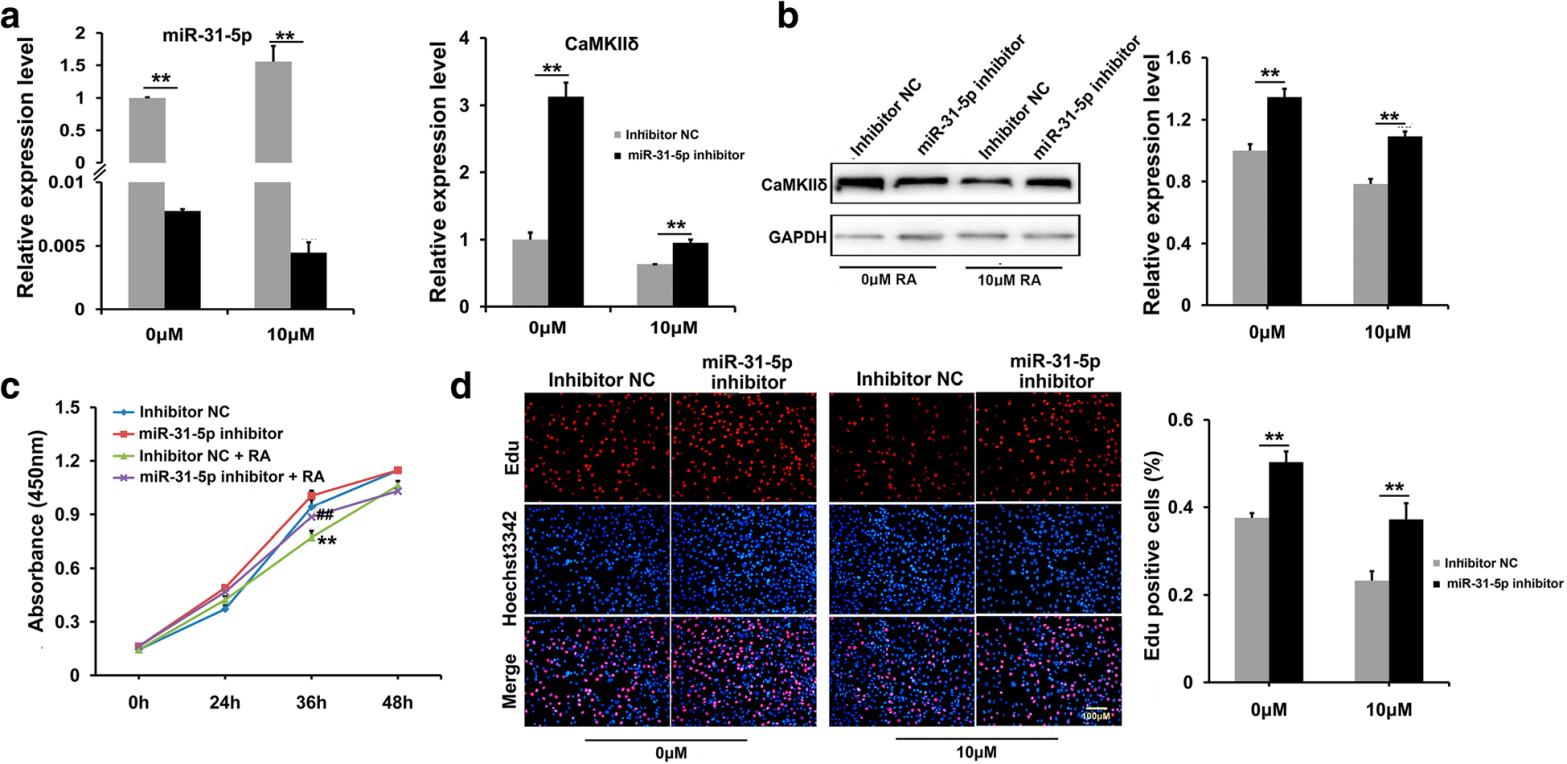
The effects on C2C12 cells proliferation by loss-of-function of *miR-31-5p*. **a** The RNA levels of *miR-31-5p* and *CamkIIδ* after 24 h transfection of *miR-31-5p* inhibitor and inhibitor NC, qRT-PCR indicated that the expression level of *miR-31-5p* was down-regulated significantly, but the *CamkIIδ* level was elevated dramatically when treated with or without RA. **b** Western blotting confirmed that CaMKIIδ was up-regulated significantly by the inhibitor in absence or presence of RA (1.35- and 1.10-folds, respectively). **c**–**d** The impacts of *miR-31-5p* inhibitor on the proliferation of C2C12 cells. CCK-8 assay indicated that the inhibition of *miR-31-5p* promoted cell proliferation and partially rescued the disturb proliferation induced by RA (**c**). Edu staining assay and statistical analysis showed that the proliferation ratio of C2C12 cells increased no matter with or without RA (**d**)

To further confirm the suppression of *miR-31-5p* on myogenic proliferation, the loss-of-function of *miR-31-5p* was simulated by transfecting *miR-31-5p* inhibitor into C2C12 myoblasts. As expected, the *miR-31-5p* transcription was extremely decreased by the inhibitor in both the absence and presence of excess RA (Fig. 3a). Along with the decreasing *miR-31-5p*, the *CamkIIδ* transcripts increased greatly in both conditions, especially when RA was absent (Fig. 3a). Correspondingly, the CaMKIIδ also increased in both the 0 and 10 μM of RA groups (Fig. 3b), though the difference in protein levels was not so large as that in the mRNA level. The absorbance curve of CCK-8 assay suggested that *miR-31-5p* inhibitor without or without RA supplement accelerated C2C12 proliferation more significantly than their corresponding controls, respectively (Fig. 3c). Consistently, EdU assay showed that no matter RA was supplemented into the medium or not, the *miR-31-5p* inhibitor was able to promote the proliferating percentage much more evidently than the controls (Fig. 3d). Thus, the *miR-31-5p* inhibitor impaired expression of *CamkIIδ* and C2C12 proliferation. Combined with the results of *miR-31-5p* mimic transfection, RA was indicated to suppress *CamkIIδ* expression by activating *miR-31-5p* transcription, which eventually inhibited the myogenic proliferation.

### RA treatment elevated *miR-31-5p*, but repressed *CamkIIδ*, *MyoD*, and *Myogenin* expression in differentiating C2C12 cells

**Fig. 4:**
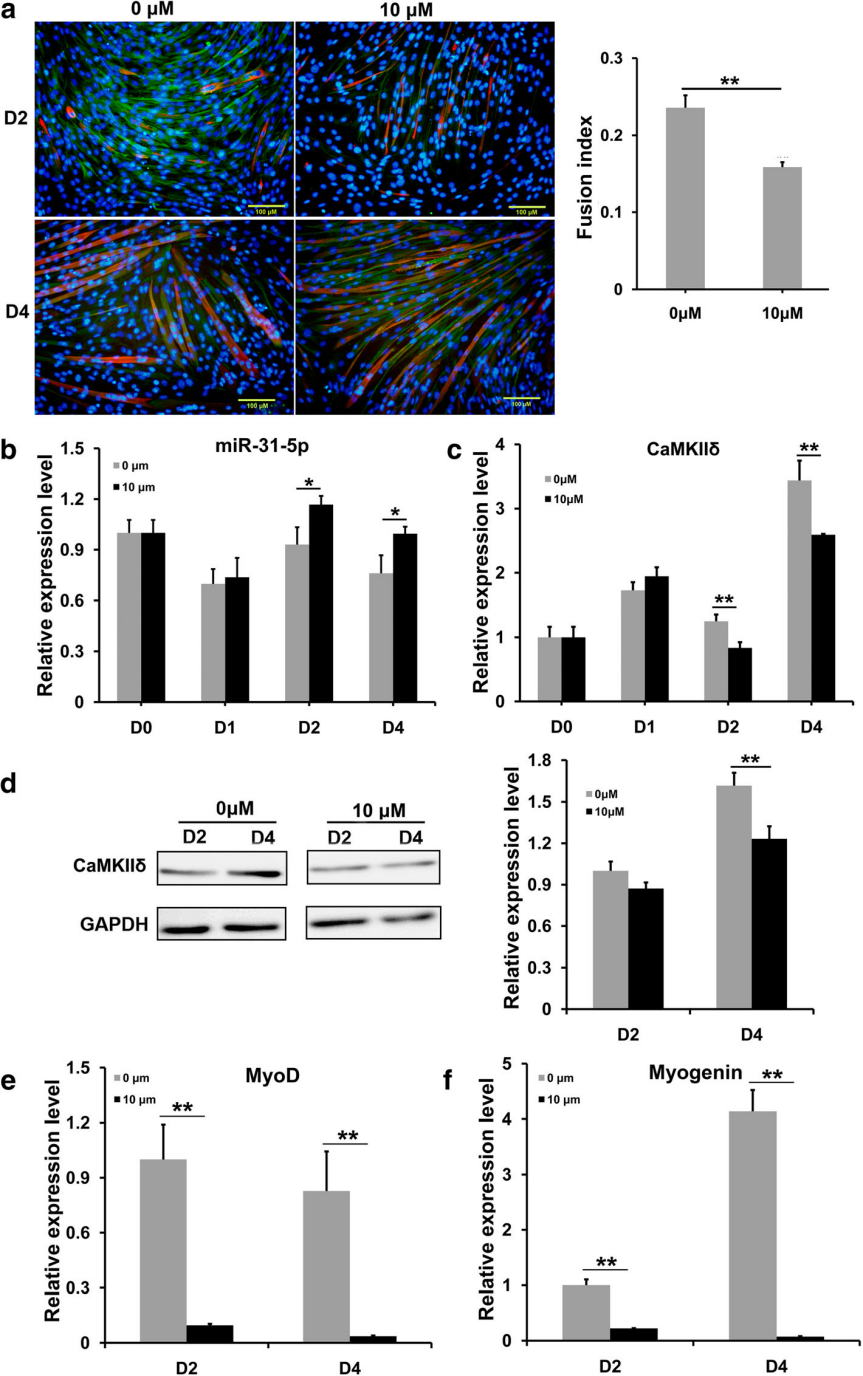
The expression of *miR-31-5p*, *CamkIIδ*, *MyoD*, and *Myogenin* in the differentiation of C2C12 cells treated by RA. **a** RA treatment impaired the myogenic differentiation of C2C12 cells. After treated with 10 μM RA for 4 days in differentiation medium, immunofluorenscence showed the decrease of myosin and CaMKIIδ expression; correspondingly, the fusion of myotubes was also decreased with RA addition. **b**, **c** The effects of RA on the expression of *miR-31-5p* and *CamkIIδ* in the differentiation process of C2C12 cells. The relative expression of *miR-31-5p* was evidently up-regulated, but the *CamkIIδ* expression was significantly down-regulated by 10 μM RA treatment. **d** Western blotting and statistical showed the down-regulation of CaMKIIδ, the reduction is 24.8%. **e**–**f** The effects of RA on the transcriptions of *MyoD* and *Myogenin*. Similar to *CamkIIδ*, the relative transcriptions of both *MyoD* (**e**) and *Myogenin* (**f**) were repressed by RA, compared to controls (*P < 0.05 and **P < 0.01)

In the E14.5 embryonic tongue, excess RA not only reduced the cell proliferation but also severely impaired myoblast differentiation [8]. Immunofluorescence staining of myosin disclosed that RA supplement caused C2C12 myoblasts to produce a more slender and reduced number of myotubes, as well as a notable drop of fusion index from the 2nd day of RA addition, indicating a retarded in vitro differentiation (Fig. 4a). The results of Q-PCR showed that from the 2nd day of RA addition, the transcriptions of *miR-31-5p* and *CamkIIδ* were up- and down-regulated, respectively (Fig. 4b, c). However, as a consequence of the reduced transcription, the significant decrease in *CamkIIδ* protein level was not detected until the 4th day of RA addition in Western blotting and the following statistical analysis (Fig. 4d). Similar to *CamkIIδ*, the transcriptions of *MyoD* and *Myogenin* were also down-regulated from the 2nd day of RA supplement (Fig. 4e, f). Combined with the reduced myosin protein level in the differentiating C2C12 cells in the presence of RA [28], it was suggested that RA inhibited myogenic differentiation by elevating *miR-31-5p* to target *CamkIIδ.*

### *MiR-31-5p* mimics repressed *CamkIIδ* expression in myoblast differentiation

**Fig. 5:**
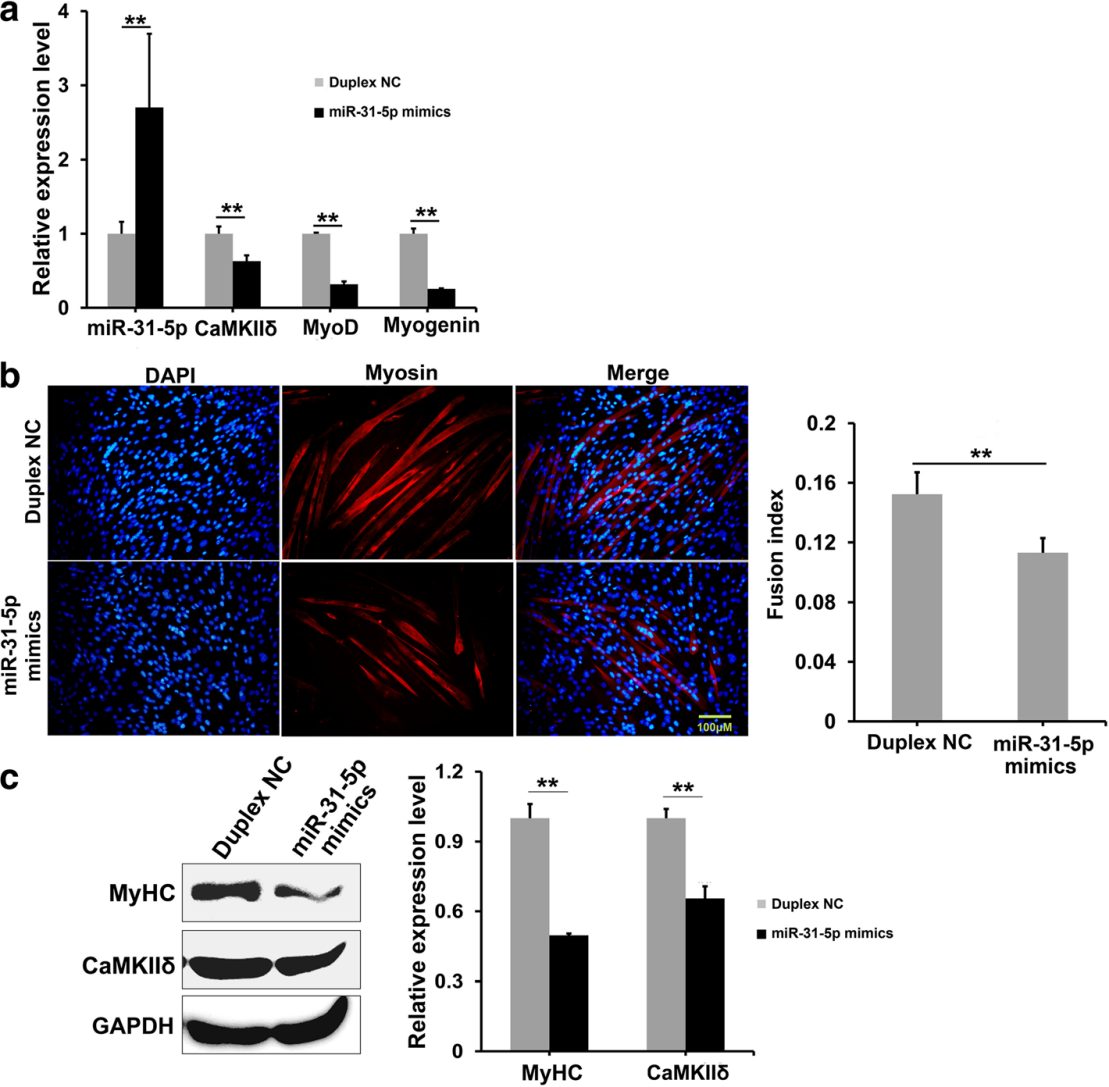
Overexpression of *miR-31-5p* repressed C2C12 cells the differentiation. **a** qRT-PCR analyzes the expression of *miR-31-5p*, *CamkIIδ*, *MyoD*, and *Mygenin*. The expression level of *miR-31-5p* increased significantly after transfaction of mimics, while the expression level of *CamkIIδ*, *MyoD*, and *Myogenin* were down-regulated dramatically. **b** Immunofluorenscence of myosin and fusion index of the myotubes. Immunostaining for myosin (*red*) showed myosin expression, and the fusion index were remarkably weakened after 4-day transfection of mimics. **c** The detection of myosin and CaMKIIδ expression by Western blotting. The protein levels of myosin and CaMKIIδ in cells transfected with mimics were decreased to 49.6 and 65.4%, respectively. (*P < 0.05 and **P < 0.01)

To explore if *miR-31-5p* suppress *CamkIIδ* expression and C2C12 differentiation, *miR-31-5p* mimics and duplex NC were transfected into the differentiating C2C12 cells. At the 4th day of transfection, Q-PCR results showed that the transcriptions of *CamkIIδ*, *MyoD*, and *Myogenin* were down-regulated remarkably in contrast to the significantly increased *miR-31-5p* level (Fig. 5a). Furthermore, *miR-31-5p* mimics reduced the number and length of myosin positive myotubes as well as the fusion index of differentiating myoblasts at the 4th day of transfection (Fig. 5b). Western blotting and the statistical analysis confirmed the significant down-regulation of endogenous myosin and CaMKIIδ by *miR-31-5p* mimics (Fig. 5c). These results demonstrated that over-expressed *miR-31-5p* could inhibit *CamkIIδ* expression and C2C12 differentiation.

### *MiR-31-5p* inhibitor partially rescued C2C12 differentiation impaired by RA

**Fig. 6:**
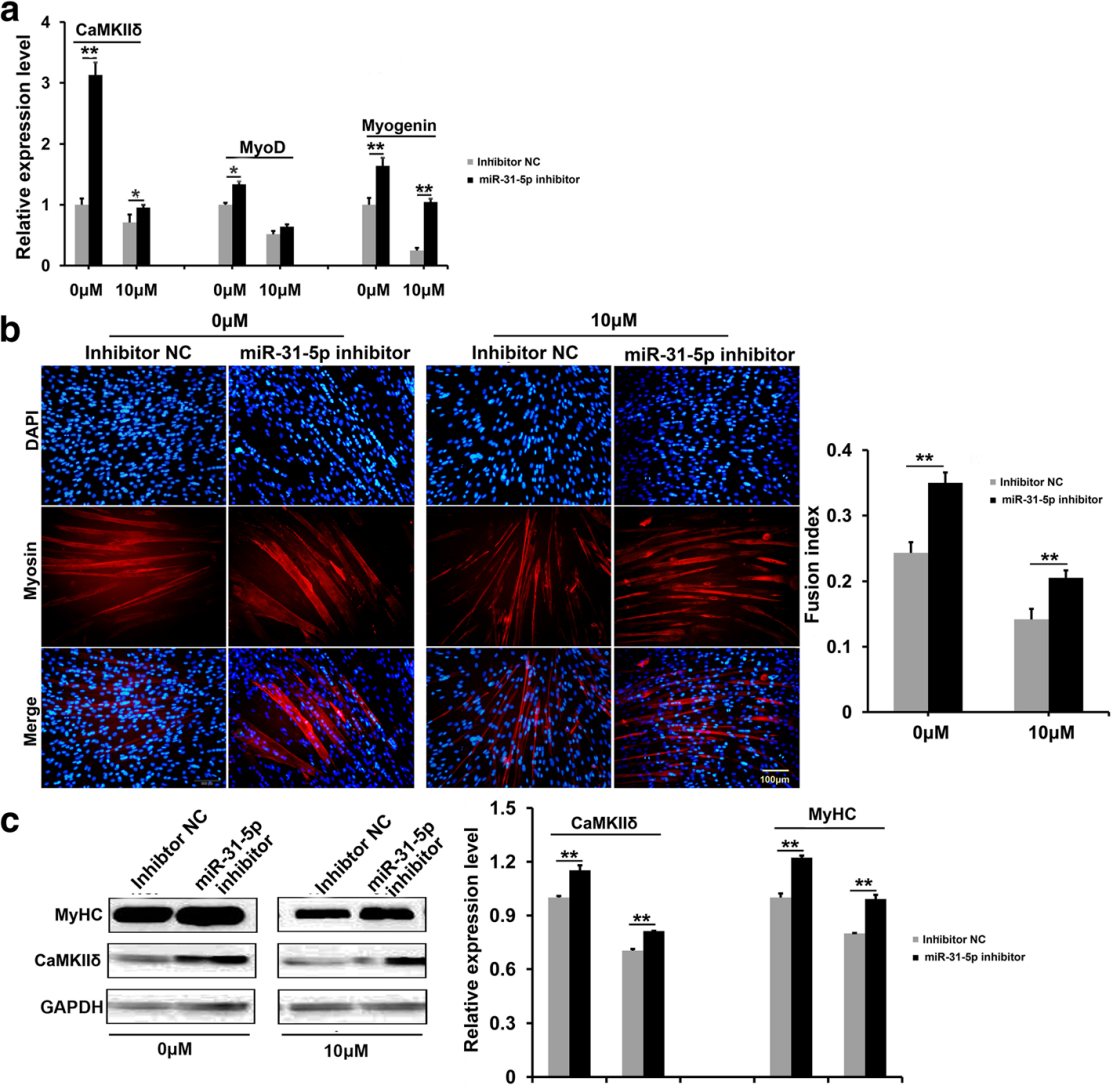
Inhibition of *miR-31-5p* promoted C2C12 cells differentiation. **a** qRT-PCR assay for expressions of *CamkIIδ*, *MyoD*, and *Myogenin* in differentiation of C2C12 cells after *miR-31-5p* inhibition. The relative expression levels of *CamkIIδ*, *MyoD*, and *Myogenin* were up-regulated without RA treatment, and in addition of RA, the *MyoD* was immune to the influence, but the levels of *CamkIIδ* and *Myogenin* were elevated. **b** Immunofluorescence of myosin and statistical analysis affected by *miR-31-5p* inhibition. *MiR-31-5p* inhibition promoted cell differentiation and myosin expression with or without RA. The fusion index confirmed the promotion of myotubes by *miR-31-5p* inhibitor. **c** Western blotting assay for detection of the effects of *miR-31-5p* inhibition on C2C12 cells differentiation. In presence of RA, the relative protein of myosin and CaMKIIδ were decreased, both of the two were remarkably up-regulated with *miR-31-5p* inhibitor than those without inhibitor. (*P < 0.05 and **P < 0.01)

The knocking down of *miR-31-5p* was conducted by the transfection of *miR-31-5p* inhibitor to thoroughly assess the role of *miR-31-5p* in myogenic differentiation and *CamkIIδ* expression. Transfection of *miR-31-5p* inhibitor could elevate transcriptions of *CamkIIδ*, *MyoD*, and *Myogenin* with or without RA (Fig. 6a). Both myosin staining and the fusion index were raised in *miR-31-5p* knock-down groups on the 4th day of transfection, revealing that *miR-31-5p* inhibitor could promote the formation of multinucleated myotubes no matter in the presence of RA or not (Fig. 6b). However, although the C2C12 myotubes in the presence of RA and *miR-31-5p* inhibitor was wider than those solely supplemented with RA, they were much thinner than those without RA supplement (Fig. 6b), which displayed a partial rescue on C2C12 differentiation by *miR-31-5p* inhibitor. Consistent with the transcription changes, the protein amounts of myosin and CaMKIIδ in both the RA absence and presence were up-regulated by *miR-31-5p* inhibitor (Fig. 6c). Thus, it was concluded that although loss-of-function of *miR-31-5p* restored the *CamkIIδ* expression, it could only partially rescued the in vitro myogenic differentiation impaired by RA.

### Knocking down of *CamkIIδ* mimicked the RA effects on C2C12 proliferation and differentiation

**Fig. 7:**
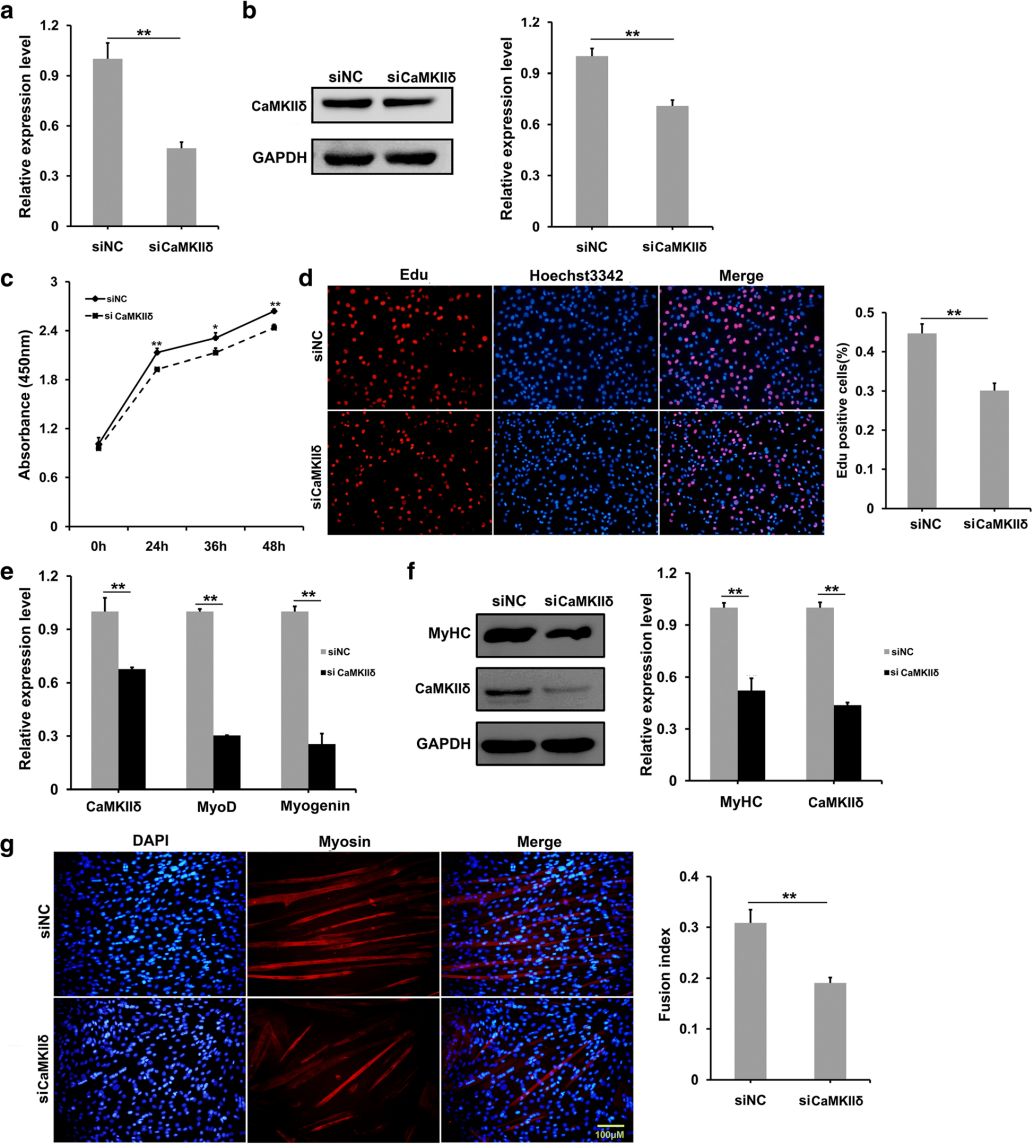
Knock-down of *CamkIIδ* repressed the proliferation and myogenic differentiation of C2C12 cells. **a**, **b** Valid knock-down effects of *CamkIIδ* by transfection of *siCamkIIδ* for 24 h. qRT-PCR (**a**) and Western blotting analysis (**b**) showed that the expression of *CamkIIδ* mRNA and protein were down-regulated 56.3 and 29.2%, respectively, after *siCamkIIδ* transfection for 24 and 48 h, respectively. **c**, **d** *siCamkIIδ*inhibited the proliferation of C2C12 cells. CCK-8 assay showed that after 48 h transfection of *siCamkIIδ*, the number of C2C12 cells increased much slower than in control group (**c**). Edu assay indicated that transfection of *siCamkIIδ*resulted in a decrease of Edu labeling cells to 60% of control (**d**). **e**, **g** *siCamkIIδ* impaired the myogenic differentiation of C2C12 cells after 4-day transfection. **e** qRT-PCR indicated the significant decrease of *CamkIIδ*, *MyoD*, and *Myogenin* by siCaMKIIδ transfection. **f** Western blotting analysis showed the dramatic down-regulation of myosin and CaMKIIδ in *siCamkIIδ* transfection group. **g** Immunofluresence of myosin staining indicated the myosin expression and fusion index reduces in *siCamkIIδ* inhibition group. (*P < 0.05 and **P < 0.01)

The transfection of *siCamkIIδ* was performed to test if CaMKIIδ mediated the repressed myogenic proliferation and differentiation induced by excess RA or elevated *miR-31-5p*. Q-PCR and Western blotting results confirmed that in the proliferating status, the mRNA, and protein of *CamkIIδ* reduced about 50 and 20% in *siCamkIIδ*-transfecting C2C12 groups, respectively, compared to the C2C12 cells transfected with siNC (Fig. 7a, b). *SiCamkIIδ* decelerated the proliferation speed of C2C12 in CCK-8 assay (Fig. 7c) and resulted in a lower proliferating percentage in EdU Assay compared with siNC (Fig. 7d). Additionally, *siCamkIIδ* transfection also remarkably suppressed the transcriptions of *CamkIIδ*, *MyoD*, and *Myogenin* in the differentiating C2C12 cells at the 4th day of transfection (Fig. 7e). Consistently, the down-regulated protein levels of CaMKIIδ and myosin in Western blotting at the identical stage implicated a suppression on C2C12 differentiation by *siCamkIIδ* (Fig. 7f). Finally, the reduction of myosin staining and fusion index at the 4th day of transfection during C2C12 differentiation in the *siCamkIIδ*-transfecting C2C12 cells convinced that *siCamkIIδ* impaired the C2C12 differentiation (Fig. 7g). Collectively, the interference on *CamkIIδ* was able to mediate the RA suppression on C2C12 proliferation and differentiation.

### Over-expression of *CamkIIδ* rescued C2C12 proliferation and differentiation by RA

**Fig. 8:**
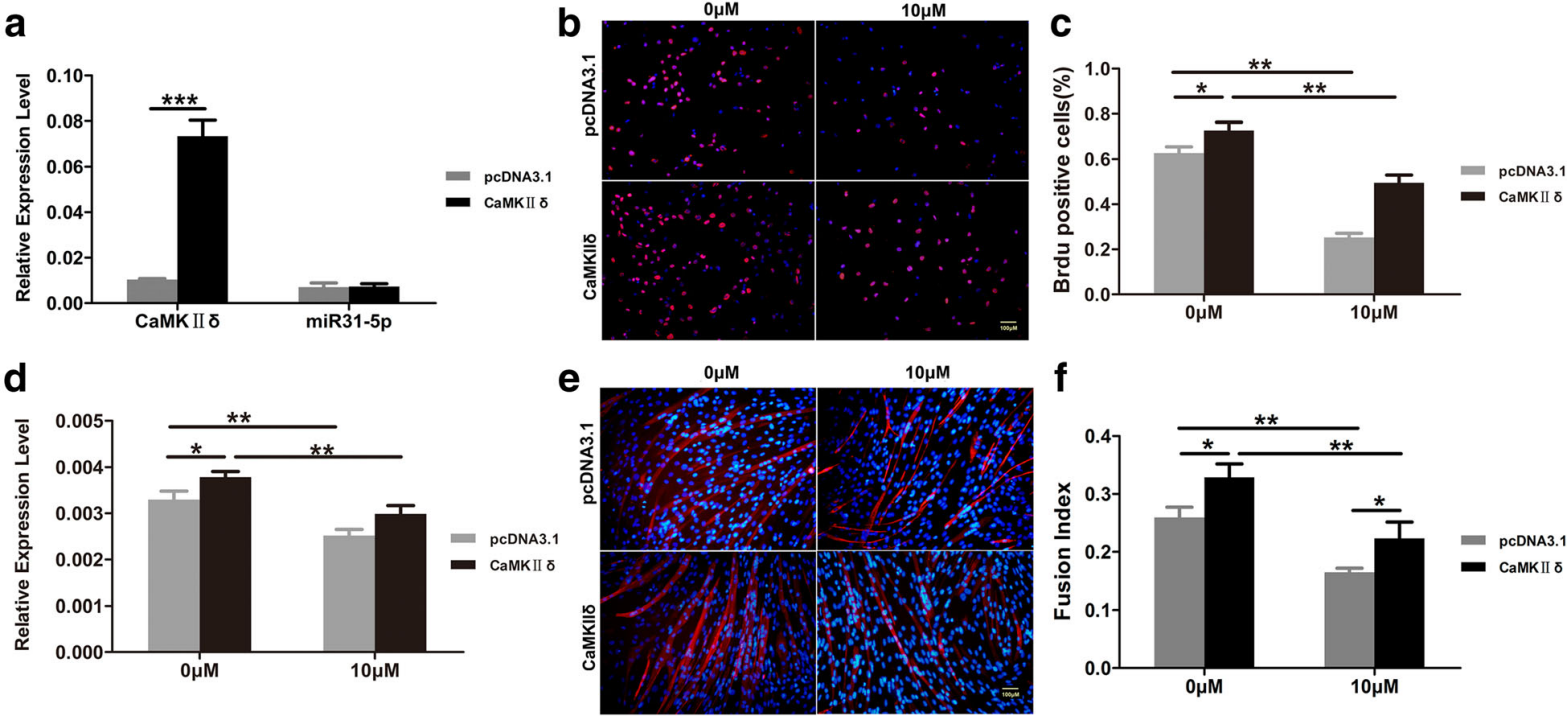
Over-expression of *CamkIIδ* with or without RA in C2C12 cells. **a**–**c** Over-expression of *CamkIIδ*in proliferating C2C12 cells. Q-PCR showed that *CamkIIδ* expression was up-regulated obviously in C2C12 myoblasts transfected with *CamkIIδ* than in cells transfected with control plasmid. On the contrary, the expression of *miR-31-5p* was not influenced by the transfection of *CamkIIδ* (**a**). Immunofluorescence of BrdU labeling of C2C12 myoblasts transfected with control plasmid, C2C12 myoblasts with excessRA and control plasmid, C2C12 myoblasts transfected by CaMKIIδ, and C2C12 myoblaststransfected by CaMKIIδwith RA (**b**). The statistical analysis of BrdU positive cells of proliferating C2C12 cells indicated that transfection of *CamkIIδ*improved the proliferation of C2C12 cells and could overcome the effects of RA on C2C12 proliferation, compared with the C2C12 transfected only by control plasmid and supplemented withRA (**c**). **d**–**f** Over-expression of *CamkIIδ*in differentiating C2C12 cells. Q-PCR indicated that the expression of *CamkIIδ* in differentiating C2C12 cells was increased with or without RA (**d**). Immunofluorescence of myosin staining of C2C12 myoblasts transfected with control plasmid, C2C12 myoblasts supplemented with RA and control plasmid, C2C12 myoblasts transfected by *CamkIIδ* without RA, and C2C12 myoblasts transfected by *CamkIIδ*with RA (**e**). The statistics of fusion index of all differentiating C2C12 groups showed that the differentiation of the C2C12 cells transfected by *CamkIIδ* and with excess RA was significantly improved compared with the C2C12 only supplemented with RA, it was still much lower than the control group and the C2C12 transfected only *CamkIIδ* (**f**). (*P < 0.05 and **P < 0.01)

To further verify the role of CaMKIIδ during C2C12 proliferation and differentiation when treated with RA, *CamkIIδ* was over-expressed in C2C12 cells in either absence or presence of RA. *CamkIIδ* over-expression has no influence on the expression of *miR-31-5p* in the proliferating C2C12 myoblasts (Fig. 8a). Compared with control, *CamkIIδ* over-expression improved the proliferation of C2C12 myoblasts and partly rescued the proliferation of C2C12 myoblasts even when excess RA was supplemented (Fig. 8b, c). Interestingly, in the differentiating C2C12 myoblasts, the transfection of *CamkIIδ* vector not only raised the *CamkIIδ* transcription in the absence of RA but also overcame the inhibition of RA on *CamkIIδ*even at the 4th day of RA addition (Fig. 8d). Consequently, the formation of myotube and fusion index of differentiating C2C12 cells were also improved in the over-expression of *CamkIIδ*, no matter RA is present or not (Fig. 8e, f). These results indicated that *CamkIIδ* over-expression could partly rescue the C2C12 proliferation and differentiation disrupted by RA.

## Discussion

### C2C12 myoblast is supposed to recapitulate RA suppression on *CamkIIδ* through miRs in embryonic tongue

The RA-induced tongue deformity was characterized by the hypolastic and deranged myofibrils along with the reduced proliferation and *myosin* expression [8]. Wnt5a/CaMKIIδ signaling pathway was implicated to mediate the teratogenic effects of RA in tongue deformity. However, how RA controls the activity of Wnt5a/CaMKIIδ signaling pathway remains unknown. A series of non-muscle specific and rich miRNAs, including *miR-27b-3p* and *miR-31-5p*, were screened out by microRNA microarray in the deformed tongue [28]. Thus, it was taken into account that the non-muscle specific and rich miRNAs mediated the RA impacts on tongue myogenesis.

Surprisingly, *miR-27b-3p* and *miR-31-5p*, which were down-regulated in the RA-treated tongue, were predicted to target *DNTA* and *CamkIIδ*, respectively, by bioinformatics analysis and luciferase reporter assay. Even though, the concern on the tissue specificity of the miRNA expression still arose because the samples for microarray and Q-PCR were the embryonic tongues, which were composed of mesoderm-derived muscle, neural crest-derived mesenchyme, and ectoderm-derived mucosa. The reciprocal interactions among tongue tissues have been established [29, 30], making the possibility that the defect in one tissue may result from the genetic alterations in another tissue. Since miRs bind to target mRNAs intracellularly and *CamkIIδ* and *DTNA* were specifically expressed in myoblasts, the *miR-27b-3p* and *miR-31-5p* activated by RA were supposed to be also confined to tongue myoblasts. The other concern in this study is the exclusion of influences from non-muscle tissues on tongue myoblasts. Although CaMKIIδ is believed to be activated by Wnt5a, the source of Wnt5a in developing tongue remains to be elucidated. As a secreted growth factor, Wnt5a can diffuse in the extracellular space and signal to cells more than myoblasts. Therefore, manipulation on *miR-31-5p* and *CamkIIδ*, rather than Wnt5a, in the C2C12 myoblasts provides an easy and reliable in vitro approach to examine our hypothesis that *miR-31-5p* mediates the RA-induced suppression on *CamkIIδ*.

### *MiR-31-5p* mediates the RA-induced suppression on *CamkIIδ* in myoblasts

Although in the RA-treated embryonic tongue, the elevated *miR-31-5p* expression was correlated with the reduced *CamkIIδ* expression, the causal relationship between them requires direct evidences. In the cultured C2C12 myoblasts, RA supplement successfully stimulated *miR-31-5p* expression and suppressed *CamkIIδ* expression as well as the impaired proliferation and differentiation, which mimic the molecular changes in the myoblasts of the RA-treated tongue. In our in vitro model, over-expression of *miR-31-5p* was able to repress *CamkIIδ* expression in both the proliferation and differentiation phases even without RA addition, verifying that *miR-31-5p* really targeted *CamkIIδ* in the C2C12 cells. On the other hand, the inhibition on *miR-31-5p* solely promoted the *CamkIIδ* expression, C2C12 proliferation and differentiation, confirmed the suppression on *CamkIIδ* by *miR-31-5p*. Taken together, we conclude that *miR-31-5p* activated by excess RA mediates the suppression on *CamkIIδ*. However, we have to point out that the *miR-31-5p* may be not the only way of RA to regulate *CamkIIδ* in the proliferation and differentiation status because the increase of *CamkIIδ* transcription by *miR-31-5p* inhibitor in the presence of RA was not so large as that in the absence of RA. Moreover, RA could exert influence on myogenic proliferation and differentiation through multiple effectors more than *miR-31-5p* and *CamkIIδ* [28]. That is why inhibition on *miR-31-5p* or *CamkIIδ* only partially rescued the differentiation of C2C12 cells.

### The role of Wnt5a/Ca^2+^ pathway and CaMKIIδ in the myoblast proliferation and differentiation

Our latest study indicated that RA-induced *miR-27b-3p* impaired the C2C12 proliferation and differentiation by suppressing *DTNA* [28]. However, not all the defects resulting from RA administration were compensated by manipulation on *miR-27b-3p*, so we explored the role of *miR-31-5p* played in the RA-induced tongue deformity. Because the inhibition on *miR-31-5p* in the RA presence only partially rescues C2C12 differentiation, we speculate that *miR-27b-3p* and *miR-31-5p* are contributed to myogenic differentiation through different mechanisms and cannot compensate for each other.

CaMKIIδ is activated by Ca^2+^ entry and acts as a component in the non-canoncial Wnt/Ca^2+^ pathway [31, 32]. As the activator of CaMKIIδ, the expression and role of Wnt5a in the developing tongue muscle are not clarified. Due to the activation of *miR27b-3p* and *miR31-5p*, instead of the muscle-specific and rich miRs in the embryonic tongue by RA, it is in the later stage of myogenic proliferation and differentiation, as opposed of the early myogenesis, that DTNA, CaMKIIδ, and Wnt5a/Ca^2+^ pathway most likely play the essential roles. We will concentrate our further investigation to explore these aspects.

## Conclusions

RA induced *miR-31-5p* inhibits the proliferation and differentiation of C2C12 cells by suppressing *CamkIIδ* expression, which recapitulates the in vivo mechanism impairing tongue myogenesis by excess RA.

## Additional files

Additional file 1: Table S1. Designed primer sequences. Note: F: foreword primer; R: reverse primer. (DOC 29 kb)

Additional file 2: Table S2. Sequences of RNA oligonucleotides used in this study. (DOC 29 kb)

Additional file 3: Table S3. The oligonucleotides’ sequences are listed. (DOC 26 kb)

## Abbreviations

CaMKII: Calcium/calmodulin-dependent protein kinase II
CCK-8: Cell Counting Kit-8
DM: Differentiation medium
EdU: 5-Ethynyl-2′-deoxyuridine
GM: Growth medium
miRs: MicroRNAs
MRFs: Myogenic regulatory factors
RA: Retinoic acid
RARE: RA response element
RARs: RA receptors
RXRs: Retinoid X receptors

## References

[1] Cunningham TJ, Duester G (2015). Mechanisms of retinoic acid signalling and its roles in organ and limb development. Nat Rev Mol Cell Biol.

[2] Beatrice Desvergne. RXR: From partnership to leadership in metabolic regulations. In. Gerald Litwack, Toluca Lake,editors. Vitamins and hormones. MArquis One: Elsevier Inc,2007. p4-10. 10.1016/S0083-6729(06)75001-4 17368310

[3] Soprano DR, Soprano KJ (1995). Retinoids as teratogens. Annu Rev Nutr.

[4] Rothman KJ, Moore LL, Singer MR, Nguyen US, Mannino S, Milunsky A (1995). Teratogenicity of high vitamin A intake. N Engl J Med.

[5] Shenefelt RE (1972). Morphogenesis of malformations in hamsters caused by retinoic acid: relation to dose and stage at treatment. Teratology.

[6] Xiao J, Zhu E-x, Nagatsuka H, Gunduz M, Li C, Minoo P, Nagai N (2005). Wnt5a gene plays a role in mouse embryonic orofacial development. J Hard Tissue Biol.

[7] Xiao J, Cong W, Wang R, Wang B, Wang F, Zhu E-x, Hu H, Katase N, Nagatsuka H (2007). The study of palatal cell proliferation and apoptosis in retinoic acidinduced mouse cleft palate varied with different developmental stage. J Hard Tissue Biol.

[8] Cong W, Liu B, Liu S, Sun M, Liu H, Yang Y, Wang R, Xiao J (2014). Implications of the Wnt5a/CaMKII pathway in retinoic acid-induced myogenic tongue abnormalities of developing mice. Sci Rep.

[9] Swaminathan PD, Purohit A, Hund TJ, Anderson ME (2012). Calmodulin-dependent protein kinase II: linking heart failure and arrhythmias. Circ Res.

[10] McKinsey TA, Zhang CL, Lu J, Olson EN (2000). Signal-dependent nuclear export of a histone deacetylase regulates muscle differentiation. Nature.

[11] Backs J, Backs T, Bezprozvannaya S, McKinsey TA, Olson EN (2008). Histone deacetylase 5 acquires calcium/calmodulin-dependent kinase II responsiveness by oligomerization with histone deacetylase 4. Mol Cell Biol.

[12] Krell MJ, Kline EM, Bates ER, Hodgson JM, Dilworth LR, Laufer N, Vogel RA, Pitt B (1986). Intermittent, ambulatory dobutamine infusions in patients with severe congestive heart failure. Am Heart J.

[13] Zhang T, Maier LS, Dalton ND, Miyamoto S, Ross J, Bers DM, Brown JH (2003). The δC isoform of CaMKII is activated in cardiac hypertrophy and induces dilated cardiomyopathy and heart failure. Circ Res.

[14] Chen X, Nakayama H, Zhang X, Ai X, Harris DM, Tang M, Zhang H, Szeto C, Stockbower K, Berretta RM, Eckhart AD, Koch WJ, Molkentin JD, Houser SR (2011). Calcium influx through Cav1.2 is a proximal signal for pathological cardiomyocyte hypertrophy. J Mol Cell Cardiol.

[15] Awad S, Al-Haffar KM, Marashly Q, Quijada P, Kunhi M, Al-Yacoub N, Wade FS, Mohammed SF, Al-Dayel F, Sutherland G, Assiri A, Sussman M, Bers D, Al-Habeeb W, Poizat C (2015). Control of histone H3 phosphorylation by CaMKIIδ in response to haemodynamic cardiac stress. J Pathol.

[16] Mishra S, Gray CB, Miyamoto S, Bers DM, Brown JH (2011). Location matters: clarifying the concept of nuclear and cytosolic CaMKII subtypes. Circ Res.

[17] Sharma A, Nguyen H, Geng C, Hinman MN, Luo G, Lou H (2014). Calcium-mediated histone modifications regulate alternative splicing in cardiomyocytes. Proc Natl Acad Sci U S A.

[18] Erickson JR, He BJ, Grumbach IM, Anderson ME (2011). CaMKII in the cardiovascular system: sensing redox states. Physiol Rev.

[19] Sweetman D, Goljanek K, Rathjen T, Oustanina S, Braun T, Dalmay T, Münsterberg A (2008). Specific requirements of MRFs for the expression of muscle specific microRNAs, miR-1, miR-206 and miR-133. Dev Biol.

[20] Horak M, Novak J, Bienertova-Vasku J (2016). Muscle-specific microRNAs in skeletal muscle development. Dev Biol.

[21] Feng Y, Cao JH, Li XY, Zhao SH (2011). Inhibition of miR-214 expression represses proliferation and differentiation of C2C12 myoblasts. Cell Biochem Funct.

[22] Luo W, Wu H, Ye Y, Li Z, Hao S, Kong L, Zheng X, Lin S, Nie Q, Zhang X (2014). The transient expression of miR-203 and its inhibiting effects on skeletal muscle cell proliferation and differentiation. Cell Death Dis.

[23] Kim JO, Song DW, Kwon EJ, Hong SE, Song HK, Min CK (2015). Kim do H. miR-185 plays an anti-hypertrophic role in the heart via multiple targets in the calcium-signaling pathways. PLoS One.

[24] Krist B, Florczyk U, Pietraszek-Gremplewicz K, Józkowicz A, Dulak J (2015). The role of miR-378a in metabolism, angiogenesis, and muscle biology. Int J Endocrinol.

[25] Luo W, Nie Q, Zhang X (2013). MicroRNAs involved in skeletal muscle differentiation. J Genet Genomics.

[26] Diniz GP, Wang DZ (2016). Regulation of skeletal muscle by microRNAs. Compr Physiol.

[27] Liu B, Li N, Jiang Y, Liu C, Ma L, Cong W, Xiao J (2016). Effects of excessive retinoic acid on C2C12 myogenesis. J Hard Tissue Biol.

[28] Li N, Tang Y, Liu B (2016). Retinoid acid-induced microRNA-27b-3p impairs C2C12 myoblast proliferation and differentiation by suppressing α-dystrobrevin. Exp Cell Res.

[29] Shuler CF, Dalrymple KR (2001). Molecular regulation of tongue and craniofacial muscle differentiation. Crit Rev Oral Biol Med.

[30] Parada C, Han D, Chai Y (2012). Molecular and cellular regulatory mechanisms of tongue myogenesis. J Dent Res.

[31] Kühl M, Sheldahl LC, Park M, Miller JR, Moon RT (2000). The Wnt/Ca2+ pathway: a new vertebrate Wnt signaling pathway takes shape. Trends Genet.

[32] Wang Q, Symes AJ, Kane CA, Freeman A, Nariculam J, Munson P, Thrasivoulou C, Masters JR, Ahmed A (2010). A novel role for Wnt/Ca2+ signaling in actin cytoskeleton remodeling and cell motility in prostate cancer. PLoS One.

