# Supplementary material for: Retinoid acid-induced microRNA-31-5p suppresses myogenic proliferation and differentiation by targeting CamkIIδ
Source: Skelet Muscle. 2017 May 11;7:8. doi: 10.1186/s13395-017-0126-x (PMC5437717; doi:10.1186/s13395-017-0126-x)
Supplement: Supplementary file 1 — Designed primer sequences. Note: F: foreword primer; R: reverse primer. (DOC 29 kb) [file 13395_2017_126_MOESM1_ESM.doc]

**Additional file 1 Table S1. Designed primer sequences.**

| Name | Accession No. | 5’-3’ |
| --- | --- | --- |
| *GAPDH* | NM_008084.2 | F:TGTGTCCGTCGTGGATCTGA  R:TTGCTGTTGAAGTCGCAGGAG |
| *CamkIIδ* | NM_001025438.1 | F: AGAAGTTCAAGGCGACCAGCA  R:GGGTATCCCACCAGCAAGATGTAG |
| *Myod* | NM_010866.2 | F:CGTGATAGATAAGTCTGGAGCTGG  R:TGCTGCTGCAGTCGATCTCTCA |
| *Myogenin* | NM_031189.2 | F: CCAGTGAATGCAACTCCCACA  R:ATGGACGTAAGGGAGTGCAGATT |

Note: F: foreword primer; R: reverse primer.
