# Supplementary material for: Retinoid acid-induced microRNA-31-5p suppresses myogenic proliferation and differentiation by targeting CamkIIδ
Source: Skelet Muscle. 2017 May 11;7:8. doi: 10.1186/s13395-017-0126-x (PMC5437717; doi:10.1186/s13395-017-0126-x)
Supplement: Supplementary file 2 — Sequences of RNA oligonucleotides used in this study. (DOC 29 kb) [file 13395_2017_126_MOESM2_ESM.doc]

**Additional file 2: Table S2.** Sequences of RNA Oligonucleotides used in this study.

| Name | Sequences | |
| --- | --- | --- |
| *MiR-31-5p* mimics | AGGCAAGAUGCUGGCAUAGCUG  GCUAUGCCAGCAUCUUGCCUU | |
| Duplex NC | UUCUCCGAACGUGCACGUTT  ACGUGACACGUUCGGAGAATT | |
| *MiR-31-5p* inhibitor | CAGCUAUGCCAGCAUCUUGCCU |  |
| Inhibitor NC | CAGUACUUUUGUGUAGUACAA |  |
| *siCamkIIδ* | GCUAGAAUCUGCCGUCUCUTT  AGAGACGGCAGAUUCUAGCTT | |
| siNC | UUCUCCGAACGUGCACGUTT  ACGUGACACGUUCGGAGAATT | |
