# Supplementary material for: Retinoid acid-induced microRNA-31-5p suppresses myogenic proliferation and differentiation by targeting CamkIIδ
Source: Skelet Muscle. 2017 May 11;7:8. doi: 10.1186/s13395-017-0126-x (PMC5437717; doi:10.1186/s13395-017-0126-x)
Supplement: Supplementary file 3 — The oligonucleotides’ sequences are listed. (DOC 26 kb) [file 13395_2017_126_MOESM3_ESM.doc]

**Additional file 3: Table S3.** The oligonucleotides’ sequences are listed.

| Oligonucleotid | Sequence |
| --- | --- |
| *pmir-GLO-CaMKIIδ-WT* | CACTGCAGTTTACCATGGGACACTGTATATATTTCTTGCCGTAATGGTAAATGACTGATT |
| *pmir-GLO-CaMKIIδ-MUT* | CACTGCAGTTTACCATGGGACAGAGTATATATTAGAACGGGTAATGGTAAATGACTGATT |
